# P-460. Clinical Presentations and Outcomes of Pediatric Mycoplasma pneumoniae: A Retrospective Chart Review

**DOI:** 10.1093/ofid/ofaf695.675

**Published:** 2026-01-11

**Authors:** Samina Bhumbra, Hannah Boyle, Muayad Allali

**Affiliations:** Indiana University School of Medicine, Indianapolis, IN; Richard M. Fairbanks School of Public Health, Indianapolis, Indiana; Indiana University School of Medicine, Indianapolis, IN

## Abstract

**Background:**

*Mycoplasma pneumoniae* causes a self-resolving illness in children, but it can also cause severe illness requiring hospitalization. Cases fell during the COVID-19 pandemic, but then surged in Asia in late 2023 and spread globally in 2024. This study aimed to characterize presentations, diagnostic challenges, and outcomes for hospitalized children with confirmed *M. pneumoniae* infections over 12 months.

**Figure:**
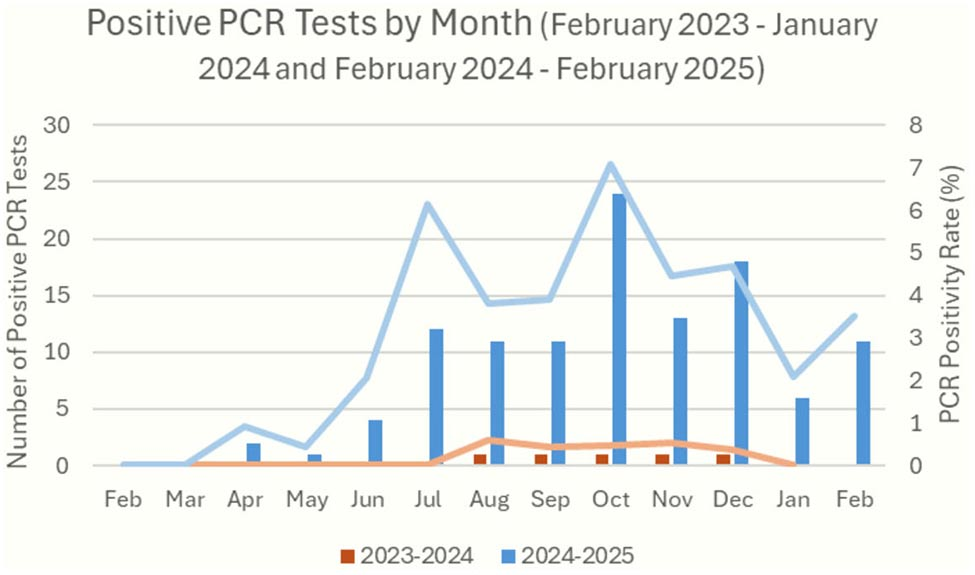

**Figure:**
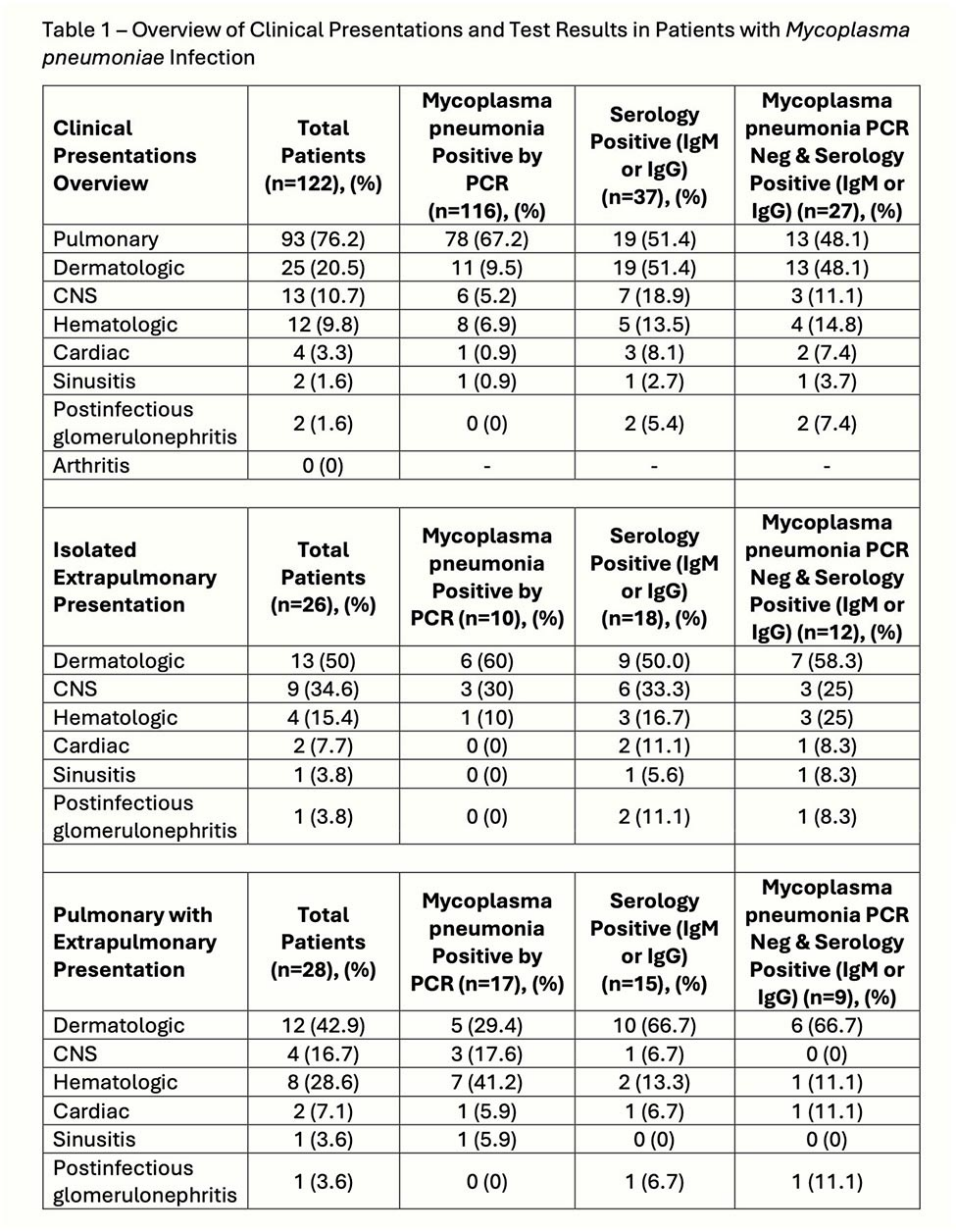

**Methods:**

A retrospective cohort study was conducted at Riley Hospital for Children of all patients admitted to pediatric units with confirmed *M. pneumoniae* infection from February 2024 to February 2025. Inclusion required a positive PCR of nasopharyngeal swab, bronchoalveolar lavage, or cerebrospinal fluid; a multiplex respiratory panel; or serology. Demographics, comorbidities, clinical presentations, diagnostic workup, treatment regimens, and outcomes were extracted to REDCap. A comparative analysis was done using data from February 2023 to January 2024.

**Results:**

Analysis identified 122 clinical encounters with 148 positive tests. There were 112/2573 (4.35%) positive molecular respiratory tests for *M. pneumoniae*, a 20.7-fold increase from the prior 12 months (5/2363; 0.21%), Figure 1. The mean age was 10.0 years (range 1 month-25 years), with 25 (20.3%) under 5 years old. An overview of presentations and *M. pneumoniae* PCR and serologic test results is available in Table 1. Notable findings include that 26 (21%) patients had isolated extrapulmonary (EP) involvement. Of isolated EP cases, 54% dermatologic and 33% CNS cases were PCR-negative but serology-positive. Viral coinfections were present in 22 cases (18%).

Intensive care unit (ICU) admission was needed for 28 cases (25%), mainly for pulmonary complications (23/28, 82%). Macrolides were given to 91 patients (75%), but 28 (23%) exhibited clinical non-response, requiring escalation to doxycycline or fluoroquinolone. One death occurred (0.8%).

**Conclusion:**

This study highlights a resurgence of pediatric *M. pneumoniae* infections in 2024–2025, Extrapulmonary manifestations, especially dermatologic and CNS, were frequent and often PCR-negative, reinforcing the need for serologic testing. Clinicians should be aware of atypical *Mycoplasma* presentations, diagnostic limitations, and treatment resistance patterns.

**Disclosures:**

All Authors: No reported disclosures

